# Strong Multivariate Relations Exist Among Milk, Oral, and Fecal Microbiomes in Mother-Infant Dyads During the First Six Months Postpartum

**DOI:** 10.1093/jn/nxy299

**Published:** 2019-05-07

**Authors:** Janet E Williams, Janae M Carrothers, Kimberly A Lackey, Nicola F Beatty, Sarah L Brooker, Haley K Peterson, Katelyn M Steinkamp, Mara A York, Bahman Shafii, William J Price, Mark A McGuire, Michelle K McGuire

**Affiliations:** 1 Department of Animal and Veterinary Sciences, University of Idaho, Moscow, ID; 2 Program in Bioinformatics and Computational Biology, University of Idaho, Moscow, ID; 3 School of Biological Sciences, Washington State University, Pullman, WA; 4 School of Family and Consumer Sciences, University of Idaho, Moscow, ID; 5 Statistical Programs, College of Agricultural and Life Sciences, University of Idaho, Moscow, ID

**Keywords:** microbiota, microbiome, human milk, breastmilk, feces, oral, maternal, infant

## Abstract

**Background:**

Neonatal gastrointestinal (GI) bacterial community structure may be related to bacterial communities of the mother, including those of her milk. However, very little is known about the diversity in and relationships among complex bacterial communities in mother-infant dyads.

**Objective:**

Our primary objective was to assess whether microbiomes of milk are associated with those of oral and fecal samples of healthy lactating women and their infants.

**Methods:**

Samples were collected 9 times from day 2 to 6 mo postpartum from 21 healthy lactating women and their infants. Milk was collected via complete breast expression, oral samples via swabs, and fecal samples from tissue (mothers) and diapers (infants). Microbiomes were characterized using high-throughput sequencing of the *16S* ribosomal RNA (rRNA) gene. Alpha and beta diversity indices were used to compare microbiomes across time and sample types. Membership and composition of microbiomes were analyzed using nonmetric multidimensional scaling and canonical correlation analysis (CCA). The contribution of various bacterial communities of the mother-infant dyad to both milk and infant fecal bacterial communities were estimated using SourceTracker2.

**Results:**

Bacterial community structures were relatively unique to each sample type. The most abundant genus in milk and maternal and infant oral samples was *Streptococcus* (47.1% ± 2.3%, 53.9% ± 1.3%, and 69.1% ± 1.8%, respectively), whereas *Bacteroides* were predominant in maternal and infant fecal microbiomes (22.9% ± 1.3% and 21.4% ± 2.4%, respectively). The milk microbiome was more similar to the infant oral microbiome than the infant fecal microbiome. However, CCA suggested strong associations between the complex microbial communities of milk and those of all other sample types collected.

**Conclusions:**

These findings suggest complex microbial interactions between breastfeeding mothers and their infants and support the hypothesis that variation in the milk microbiome may influence the infant GI microbiome.

## Introduction

High-throughput sequencing has unveiled distinct and complex consortia of microbial communities in and on the human body (e.g., 1–4), and their compositions are associated with health and disease states (5). As a mother and her breastfed infant form a unique epi-holobiont (6), their microbiomes likely interact to also influence maternal and infant health (7–14). Thus, understanding how these microbiomes are structured, maintained, and relate to each other is important.

Several studies have compared the microbiomes from various sites in the mother-infant dyad (15–28). These studies, along with the Human Microbiome Project and other studies (1–4), have reported distinct bacterial communities in and on different body sites, although some similarities also exist. For instance, milk, and infant oral and maternal oral samples are often predominately comprised of *Streptococcus* (21–23). Differences in bacterial diversity across some but not all niches have also been reported (20, 23, 27), as have changes over time. For example, the microbiomes of infant feces (16, 22, 27) and milk (20, 21, 29) vary across time, whereas maternal fecal (21, 30) and oral (21) bacterial communities appear to remain relatively stable. Although many of these studies have begun to elucidate the potential relationships among mother-infant microbiomes, a rigorous comparison of the microbiomes of milk, maternal oral and fecal samples, and infant oral and fecal samples collected from the same mother-infant dyads across several time points in the first 6 mo postpartum has yet to be conducted.

To help close this knowledge gap, we studied mother-infant dyads between birth and 6 mo postpartum. Samples included milk, oral swabs, and feces from mothers; and oral swabs and feces from their infants. Our goals were to: *1*) characterize bacterial diversity in the various sample types over time, *2*) assess the dissimilarity/similarity of the bacterial communities among the sample types over time, and *3*) assess multivariate relations among bacterial communities of the different sample types. Our overarching a priori hypothesis was that the milk microbiome would be more similar to that of infant feces than the other biological niches investigated. It is noteworthy that the composition of maternal fecal and milk microbiomes from this study have been described previously (29, 30).

## Methods

### Subjects and study design

This study was carried out as a prospective, longitudinal investigation of 21 healthy, breastfeeding women recruited in their third trimester of pregnancy from the Pullman, WA/Moscow, ID, area. Samples and data were collected on 2, 5, and 10 d (±1 d), and 1, 2, 3, 4, 5, and 6 mo (±1 d) postpartum between June 2011 and April 2012. Mothers needed to be ≥18 y old and planning to breastfeed for ≥6 mo to participate. Nonexclusive breastfeeding was not a reason for exclusion, although most infants (*n* = 11) were exclusively fed the milk of their mother until they were 3 mo of age. Most other infants (*n* = 10) were predominantly fed the milk of their mother but occasionally received fluids such as water, glucose-water, and other liquids during the first 3 mo.

### Sample collection

Methods used to collect maternal feces and milk have been described elsewhere (29, 30). Briefly, milk was collected from the same breast at each sampling time point using sterile collection kits and electric pumps, placed on ice, and transported to the laboratory where it was aliquoted and frozen at −80°C until processing. Oral samples were obtained by swabbing the dorsum of the tongue and the interior cheek surfaces using a sterile viscose-tipped swab (Sarstedt; #80.625). Oral samples were capped, stored on ice, and transported to the laboratory where swabs were either stored directly at −80°C or cut with scissors cleaned with 70% ethanol in water and transferred to tubes containing 0.5 mL sterile Tris-EDTA buffer (TE; 10 mM Tris-HCl, 1 mM EDTA, pH 8) and stored at −80°C.

Infant fecal samples were collected from a soiled diaper or from the infant's skin. In most cases, feces were immediately transferred into a sterile tube using a sterile viscose-tipped swab. In a few circumstances, caregivers froze the entire soiled diaper. If collected at home by the caregiver, samples were stored in a home freezer until they could be retrieved by study personnel (typically within 1 d). Samples collected at a university site were immediately stored at −80°C; those collected at a home or hospital site were kept on ice until they could be frozen at −80°C.

### Extraction of DNA

Extraction of DNA from maternal feces and milk has been described elsewhere (29, 30). For oral samples, if not already done prior to freezing, ends of the swabs were cut and transferred to sterile tubes containing 0.5 mL TE as described above. Swab tips were vortexed (30 sec), the liquid transferred to a new sterile tube, centrifuged (13,000 × *g* for 10 min at 4°C), the supernatant decanted, and the remaining pellet resuspended in sterile 0.5 mL TE50 (10 mM Tris-HCl, 50 mM EDTA, pH 8.0). Samples were subjected to enzymatic and physical lysis as described previously (29), and DNA was extracted using the QIAamp DNA Mini Kit (Qiagen Cat. 51,304) following the manufacturer's protocol. TE50 (0.5 mL) was used as a negative control. Extracted DNA was eluted in 50 µL of nuclease-free water (Invitrogen Cat. AM9937) and stored at −80°C until further analysis.

In most cases, DNA was extracted from infant feces by first cutting the tip of the swab and transferring it to a sterile tube with 0.5 mL TE50 as described above. When whole diapers were frozen, a soiled portion was cut and used. Soiled diaper pieces or swab tips containing feces were vigorously vortexed, the liquid transferred to a new tube, and processed using the QIAamp DNA Stool Mini Kit (Qiagen Cat. 51,504) following the manufacturer's protocol.

### Amplification and sequencing of bacterial DNA

To prepare all samples for sequencing, we used a dual-barcoded, 2-step PCR to amplify the V1-V3 hypervariable region of the bacterial *16S* ribosomal RNA (rRNA) gene. Descriptions of the primers used have been reported previously (29). All PCR procedures and reactions were conducted in a dedicated PCR hood. Negative DNA extraction controls were implemented for each set of extractions. If amplification bands were observed in these controls, extractions and/or the PCR were repeated as required.

Bacterial DNA from maternal and infant feces was amplified as described previously (30), and from milk as described by Williams and colleagues (29). The first PCR for all oral samples was conducted as described previously (29). Products from the first PCR were evaluated for quality and underwent a second PCR as described (30) with the addition of 360 GC Enhancer (ThermoFisher Scientific, Carlsbad, CA; 1.0 µL/20 µL reaction volume) and the following conditions: 94°C for 5 min; then 94°C for 30 sec, 60°C for 45 sec, and 72°C for 1.5 min for 20 cycles with a 0.5°C step-down in the annealing temp for each cycle, then 94°C for 30 sec, 50°C for 45 sec, and 72°C for 1.5 min for 10 cycles; and a final extension step of 72°C for 5 min. Samples were held at 4°C in the thermocycler until being stored at −20°C. The quality of second PCR amplicons was evaluated using a QIAxcel DNA screening cartridge (Qiagen), and DNA quantified using the Quant-iT Picogreen double stranded DNA (dsDNA) Assay Kit (Invitrogen) or Qubit dsDNA HS Assay Kit (Invitrogen).

An appropriate volume of each amplicon (containing 50 ng DNA) was pooled to create a composite sample with processing and sequencing as described previously (29). Raw DNA sequence reads were demultiplexed and processed using the custom python application dbcAmplicons (29, 31).

### Longitudinal characterization and statistical analyses of microbiomes

Individual-based rarefaction and accumulation curves were generated using the rarecurve and specaccum functions, respectively, in the vegan package (v2.4.6) in R (32, 33). Individual-based rarefaction curves and accumulation curves (not shown) suggested that the threshold for sequencing and sampling depths varied across sample types and taxonomic levels, but that we could confidently analyze data classified at the phylum level with at least 2,000 sequences and ∼50 samples. However, because we were interested in comparing our data to other previously published data (at the genus level) and generating hypotheses for future investigations with more sequencing depth, we also examined bacterial community structures at the genus level. As there was considerable variation in total sequencing read counts for each sample and across sample types, read counts were rarefied at 2,000 reads/sample prior to calculation of various indices and conducting multivariate analyses using the rrarefy function in the vegan package in R. Following rarefication, sequence read counts for each taxon were converted to relative abundance values by taking the sum of read count per taxa per sample and dividing by 2,000. Relative abundances were summarized by sample type and time postpartum.

### Diversity metrics and statistical analyses

Alpha diversity, describing “within” sample diversity (34, 35), was assessed at the genus level using richness, Simpson evenness, Pielou's J, and Shannon's diversity metrics which were calculated using the vegan package in R. Richness reflects the number of different taxa observed in a sample; Simpson and Pielou's J evenness reflect how evenly distributed the abundances of the taxa are in the community (2 evenness measures were chosen as Simpson evenness is sensitive to changes in dominance); Shannon's diversity combines richness and evenness and is sensitive to changes in abundances of the rare groups (36). Statistical analyses of diversity indices were performed in SAS version 9.3 (SAS Institute Inc.) using a generalized linear mixed model (GLMM) with time postpartum, sample type (e.g., infant feces, milk), and the time by sample type interaction. GLMM models assumed a Poisson response distribution, with participant as a random effect and an autoregressive repeated-measure structure across sampling time. When the interaction was significant, the effects of time within each sample type were assessed individually. Probability (*P*) values were adjusted for multiple comparisons using a Bonferroni correction. Significance for the specified responses was declared at *P* ≤ 0.05.

Beta diversity (“between” sample diversity) was examined using principal coordinates analysis (PCoA) using Hellinger-transformed data and nonmetric dimensional scaling (NMDS) using the Bray–Curtis dissimilarity matrix. PCoA and NMDS are both ordination techniques that visualize the similarity or dissimilarity of various bacterial communities (37). Analysis of similarity (ANOSIM; 38) and adonis (39) functions in the vegan package in R were utilized to statistically test for differences in microbial community composition among sample types. Bray–Curtis dissimilarity distance matrices were utilized in both tests, and analyses for both were conducted with 999 permutations.

To further investigate relations among bacterial communities of different sample types, we conducted canonical correlation analyses (CCAs; 40) among pairs of sample types using the relative abundances of the most abundant taxa in each sample type based on the rarefied count data. CCA allows exploration of relations between 2 multivariate sets of variables—in this case, complex microbiomes. CCA strives to identify linear combinations within one set of variables that maximizes the correlations with a linear combination within a second set of variables. CCA was utilized to expand our analyses from only exploring univariate relations between individual taxa. CCAs were computed using PROC CANCORR in SAS v9.3.

Additionally, SourceTracker2 (version 2.0.1; 41) was utilized to estimate *1*) the contribution of maternal oral, milk, and fecal (proxy for gastrointestinal [GI]), and also infant oral bacteria to the bacterial composition of the infant fecal microbiome, and *2*) the contribution of maternal fecal, oral, and infant oral bacteria to the milk microbiome. For both estimations, rarefied sequence count data were used.

Relative abundance values listed represent means ± SEM. Diversity values listed represent least square means ± SEM.

## Results

### Subject description and sample disposition

Information related to basic anthropometrics and reproductive history for all subjects at enrollment is listed in **Table 1** and has been described previously (30). On average, women were 30 ± 4 y old, weighed 64 ± 7 kg prior to pregnancy, and had 1.8 ± 1.0 children. Most samples were collected from both mother and infant at each time point, but we were unable to obtain all samples due to subject unavailability, subject noncompliance, or mishandling of the sample by study personnel. Ultimately, 181 infant fecal, 184 infant oral, 167 maternal fecal, 183 maternal oral, and 168 milk samples were obtained.

**TABLE 1:** Selected anthropometric and descriptive variables of the 21 lactating women participating in this study^1^

Age, y	30 ± 4
Height, cm	166 ± 9
Prepregnancy wt, kg	64 ± 7
Postpartum wt, kg	71 ± 9
Postpartum BMI, kg/m^2^	26.0 ± 4
Delivery mode	
Vaginal, *n*	16
Cesarean, *n*	5
Delivery location	
Hospital, *n*	19
Home, *n*	2
Parity, *n*	1.8 ± 1
Female infants, %	38
Exclusively breastfed at 3 mo, %	52

1 Values are means ± SD or unit of measure as indicated; a total of 21 women and their infants were studied.

### Sequencing summary

In total, 911 samples (some being duplicates) were sequenced yielding a total of 18,534,383 sequencing reads (range: 2 to 154,386 reads; mean ± SEM: 20,345 ± 590 reads) following demultiplexing and sequence read processing, 1,022 taxa were identified. After removing duplicates and samples with <2,000 reads, 791 samples (149 infant feces, 151 infant oral, 162 maternal feces, 182 maternal oral, and 147 milk) were used in the analyses (**Table 2**). Sequences were classified at phylum, class, order, family, and genus levels using the Ribosomal Database Project (RDP) classifier (42) and the RDP database (43). The classification of a sequence at a particular level had to meet a bootstrap confidence threshold of at least 0.50 to be classified at that taxonomic level. Although most sequences could be classified to the genus level with this threshold, some could not. For instance, 1.2% of the sequence reads in infant oral samples were identified as being in the Lactobacillales order (the 6th most abundant taxon in this sample type), but the sequences in this Lactobacillales group did not meet the bootstrap confidence threshold for lower levels and thus, were not classified with a genus taxonomic name. In this case and others like it, we decided to include Lactobacillales in the analyses to prevent the loss of information from sequences on taxa that were present in the community but could not be identified at a lower taxonomic level. After classification, the taxa in the genus-level grouping were filtered by removing taxon with a sum <40 reads total over all of the samples, a mean of <2 reads/taxon, and were present in <21 samples, resulting in a total of 366 taxa across the 5 different sample types. Due to variability in the mean number of reads among the various sample types, sample read counts were also rarefied to 2,000 reads/sample.

**TABLE 2:** Number and type of samples analyzed at each time point from the mothers and infants included in this analysis

	Day 2	Day 5	Day 10	1 mo	2 mo	3 mo	4 mo	5 mo	6 mo	Total
Infant feces	14	16	19	17	18	16	18	18	13	149
Infant oral	17	15	17	18	17	17	19	16	15	151
Maternal feces	15	19	19	19	19	20	17	17	17	162
Milk	10	18	15	20	18	17	17	16	16	147
Maternal oral	20	21	21	21	20	20	20	20	19	182
Total	76	89	91	95	92	90	91	87	80	791

### Alpha and beta diversity

**FIGURE 1:**
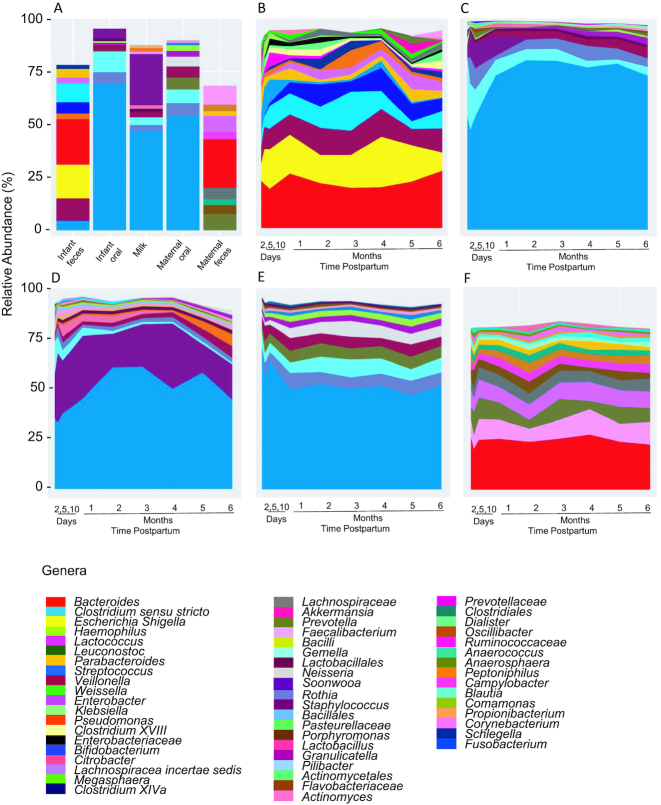
Relative abundances of (A) the top 10 taxa at the genus level for each sample type; and relative abundances across time for (B) infant fecal genus-level taxa; (C) infant oral genus-level taxa; (D) milk genus-level taxa; (E) maternal oral genus-level taxa; (F) maternal fecal genus-level taxa. The taxa represented in 1B-1F represent each sample type's most abundant taxa and correspond to taxa in Table 5.

A sample type by time interaction was evident (*P* < 0.005) for each of the diversity indices. As such, we examined the effect of time on diversity indices within each sample type and the effect of sample type across time (**Table 3**). However, after adjusting for multiple comparisons, few changes across time were noted. Microbial richness decreased from day 2 to day 10 in infant feces. The Simpson evenness index decreased in infant oral samples from day 2 and day 10 to 4 and 5 mo indicating that the relative abundances of the taxon within this community became less evenly distributed (see **Figure 1**C). With respect to Shannon diversity, the only difference observed was an increase between day 2 and 5 mo in the maternal oral swabs, indicating the bacterial communities in the mothers’ oral samples were more diverse and the membership more evenly distributed later in the postpartum period. When comparing diversity across sample types, maternal feces had greater richness (*P* < 0.0001) than infant feces and oral samples. The Shannon diversity of maternal feces was also greater (*P* < 0.008) than most sample types at each time point.

**TABLE 3:** Alpha bacterial diversity metrics (richness, Simpson evenness, Pielou's J, and Shannon diversity) during the first 6 mo postpartum in infant feces, infant oral, maternal feces, maternal oral, and milk samples^1^

	Time postpartum								
	Day 2	Day 5	Day 10	1 mo	2 mo	3 mo	4 mo	5 mo	6 mo
*Richness*									
Infant feces	42 ± 3^a^	29 ± 3^ab^	26 ± 2^b^	27 ± 2^ab^	29 ± 2^ab^	30 ± 3^ab^	34 ± 3^ab^	33 ± 3^ab^	34 ± 3^ab^
Infant oral	23 ± 2	23 ± 2	21 ± 2	20 ± 2	22 ± 2	24 ± 2	28 ± 2	28 ± 3	29 ± 3
Maternal feces	64 ± 4	65 ± 4	66 ± 4	68 ± 4	65 ± 4	63 ± 3	65 ± 4	67 ± 4	64 ± 4
Maternal oral	42 ± 3	50 ± 3	47 ± 3	56 ± 3	49 ± 3	48 ± 3	50 ± 3	57 ± 3	51 ± 3
Milk	49 ± 4	46 ± 3	50 ± 4	44 ± 3	51 ± 3	47 ± 3	47 ± 3	59 ± 4	52 ± 3
*Simpson evenness*									
Infant feces	0.09 ± 0.01	0.10 ± 0.01	0.11 ± 0.01	0.11 ± 0.01	0.11 ± 0.01	0.10 ± 0.01	0.09 ± 0.01	0.10 ± 0.01	0.12 ± 0.01
Infant oral	0.11 ± 0.01^a^	0.10 ± 0.01^ab^	0.11 ± 0.01^a^	0.10 ± 0.01^ab^	0.07 ± 0.01^ab^	0.07 ± 0.01^ab^	0.06 ± 0.01^b^	0.06 ± 0.01^b^	0.07 ± 0.01^ab^
Maternal feces	0.12 ± 0.01	0.11 ± 0.01	0.10 ± 0.01	0.12 ± 0.01	0.12 ± 0.01	0.12 ± 0.01	0.10 ± 0.01	0.12 ± 0.01	0.12 ± 0.01
Maternal oral	0.05 ± 0.01	0.06 ± 0.01	0.06 ± 0.01	0.07 ± 0.01	0.07 ± 0.01	0.08 ± 0.01	0.07 ± 0.01	0.08 ± 0.01	0.07 ± 0.01
Milk	0.08 ± 0.01	0.07 ± 0.01	0.06 ± 0.01	0.06 ± 0.01	0.05 ± 0.01	0.04 ± 0.01	0.04 ± 0.01	0.04 ± 0.01	0.07 ± 0.01
*Pielou's J*									
Infant feces	0.36 ± 0.03	0.36 ± 0.03	0.38 ± 0.02	0.43 ± 0.03	0.43 ± 0.03	0.42 ± 0.03	0.41 ± 0.03	0.45 ± 0.03	0.49 ± 0.03
Infant oral	0.32 ± 0.02	0.34 ± 0.03	0.33 ± 0.02	0.25 ± 0.02	0.24 ± 0.02	0.26 ± 0.02	0.30 ± 0.02	0.28 ± 0.02	0.33 ± 0.03
Maternal feces	0.63 ± 0.03	0.60 ± 0.03	0.59 ± 0.03	0.63 ± 0.03	0.63 ± 0.03	0.62 ± 0.03	0.61 ± 0.03	0.64 ± 0.03	0.62 ± 0.03
Maternal oral	0.37 ± 0.02	0.43 ± 0.03	0.40 ± 0.02	0.49 ± 0.03	0.45 ± 0.03	0.46 ± 0.03	0.46 ± 0.03	0.50 ± 0.03	0.46 ± 0.03
Milk	0.43 ± 0.03	0.42 ± 0.03	0.39 ± 0.03	0.34 ± 0.02	0.35 ± 0.02	0.31 ± 0.02	0.30 ± 0.02	0.38 ± 0.03	0.42 ± 0.03
*Shannon diversity*									
Infant feces	1.4 ± 0.1	1.2 ± 0.1	1.2 ± 0.1	1.4 ± 0.1	1.5 ± 0.1	1.5 ± 0.1	1.4 ± 0.1	1.6 ± 0.1	1.7 ± 0.1
Infant oral	1.0 ± 0.1	1.1 ± 0.1	1.0 ± 0.1	0.8 ± 0.1	0.7 ± 0.1	0.8 ± 0.1	1.0 ± 0.1	0.9 ± 0.1	1.1 ± 0.1
Maternal feces	2.6 ± 0.2	2.5 ± 0.1	2.5 ± 0.1	2.6 ± 0.1	2.6 ± 0.1	2.6 ± 0.1	2.5 ± 0.1	2.7 ± 0.2	2.6 ± 0.1
Maternal oral	1.4 ± 0.1^b^	1.7 ± 0.1^ab^	1.5 ± 0.1^ab^	2.0 ± 0.1^ab^	1.7 ± 0.1^ab^	1.8 ± 0.1^ab^	1.8 ± 0.1^ab^	2.0 ± 0.1^a^	1.8 ± 0.1^ab^
Milk	1.7 ± 0.2	1.6 ± 0.1	1.5 ± 0.1	1.3 ± 0.1	1.4 ± 0.1	1.2 ± 0.1	1.2 ± 0.1	1.5 ± 0.1	1.7 ± 0.1

1 Values are least square means ± SEM; infant feces, *n* = 149; infant oral, *n* = 151; maternal feces, *n* = 162; maternal oral, *n* = 182; milk, *n* = 147. Values within a row not sharing a common superscript differ (*P* < 0.05). Diversity indices were calculated using the rarefied genus-level count data. Richness: higher number, more taxa present; Simpson evenness: higher number, community is more evenly distributed; Pielou's J: higher number, community is more evenly distributed; Shannon diversity: higher number, higher diversity.

### Characterization of the most abundant bacterial taxa

Average relative abundances of the 4 most abundant phyla are presented in **Table 4**. Infant oral, maternal oral, and milk microbial communities were predominated by Firmicutes; maternal feces by Firmicutes and Bacteroidetes; and infant feces was characterized by a relatively even distribution of Firmicutes, Bacteroidetes, and Proteobacteria. The average relative abundances of the taxa identified at the genus level (or lowest level of classification) and constituting 1 of the 10 most abundant taxa in at least one time point for that sample type are listed in **Table 5** and graphically represented in Figure 1A–E. Relative abundances for the 20 most abundant taxa classified at the genus level or lowest level of classification in each sample type are provided in **Supplemental Table 1**. Although each sample type had a unique bacterial community composition, there were several noteworthy similarities. For example, infant and maternal oral swabs were both predominated by *Streptococcus* (69.1% ± 1.8% and 53.9% ± 1.3%, respectively); whereas the most abundant genus in infant and maternal feces was *Bacteroides* (21.4% ± 2.4% and 22.9% ± 1.3%, respectively). The 5 most abundant genera in both milk and infant oral samples were *Streptococcus, Staphylococcus, Gemella, Rothia*, and *Veillonella*. With the exception of *Staphylococcus*, these genera were also the most abundant in maternal oral samples.

**TABLE 4:** Overall relative abundances (%) of the 4 most abundant phyla averaged across all time points for infant feces and oral samples, maternal feces, oral, and milk samples^1^

Sample type	Phyla	Relative abundance (%)
Infant feces	Firmicutes	39.3 ± 2.6
	Proteobacteria	27.6 ± 2.1
	Bacteroidetes	26.4 ± 2.1
	Actinobacteria	6.1 ± 0.9
Infant oral	Firmicutes	89.3 ± 1.4
	Actinobacteria	6.6 ± 1.0
	Bacteroidetes	2.1 ± 0.5
	Proteobacteria	1.8 ± 0.6
Maternal feces	Firmicutes	52.3 ± 1.1
	Bacteroidetes	41.7 ± 1.1
	Proteobacteria	3.3 ± 0.3
	Actinobacteria	1.2 ± 0.2
Maternal oral	Firmicutes	71.2 ± 1.1
	Proteobacteria	9.9 ± 0.6
	Bacteroidetes	8.3 ± 0.6
	Actinobacteria	8.0 ± 0.5
Milk	Firmicutes	86.9 ± 1.2
	Actinobacteria	6.8 ± 0.8
	Proteobacteria	4.2 ± 0.8
	Bacteroidetes	1.8 ± 0.3

1 Values are means ± SEM; infant feces, *n* = 149; infant oral, *n* = 151; maternal feces, *n* = 162; maternal oral, *n* = 182; milk, *n* = 147.

**TABLE 5:** Mean relative abundances of the aggregated list of top 10 most abundant genera (or next highest characterizable taxa) from each timepoint in infant feces, infant oral, maternal feces, maternal oral, and milk samples from 2 d to 6 mo postpartum^1^

Infant feces	%	Infant oral	%	Maternal feces	%	Maternal oral	%	Milk	%
*Bacteroides*	21.4 ± 2.4	*Streptococcus*	69.1 ± 1.8	*Bacteroides*	22.9 ± 1.3	*Streptococcus*	53.9 ± 1.3	*Streptococcus*	47.1 ± 2.3
*Escherichia/Shigella*	16.0 ± 2.0	*Gemella*	9.5 ± 1.3	*Faecalibacterium*	8.8 ± 0.6	*Rothia*	6.3 ± 0.5	*Staphylococcus*	24.1 ± 2.2
*Veillonella*	10.5 ± 1.4	*Rothia*	5.7 ± 1.0	*Prevotella*	7.6 ± 1.0	*Gemella*	6.2 ± 0.5	*Gemella*	3.6 ± 0.8
*Clostridium sensu stricto*	9.0 ± 1.6	*Staphylococcus*	4.1 ± 0.9	*Lachnospiracea incertae sedis*	7.6 ± 0.4	*Prevotella*	5.8 ± 0.5	*Rothia*	2.6 ± 0.5
*Bifidobacterium*	5.4 ± 0.9	*Veillonella*	2.6 ± 0.3	*Lachnospiraceae*	5.5 ± 0.3	*Veillonella*	5.3 ± 0.3	*Veillonella*	2.5 ± 0.3
*Streptococcus*	4.6 ± 1.2	*Lactobacillales*	1.2 ± 0.0	*Porphyromonas*	4.2 ± 0.6	*Neisseria*	4.5 ± 0.4	*Lactobacillus*	1.7 ± 0.7
*Parabacteroides*	4.0 ± 1.0	*Granulicatella*	1.1 ± 0.2	*Ruminococcaceae*	3.4 ± 0.2	*Granulicatella*	2.7 ± 0.2	*Pseudomonas*	1.7 ± 0.6
*Lachnospiracea incertae sedis*	2.8 ± 0.7	*Haemophilus*	0.9 ± 0.4	*Oscillibacter*	3.2 ± 0.3	*Haemophilus*	2.7 ± 0.2	Lactobacillales	1.7 ± 0.1
*Pseudomonas*	2.7 ± 0.5	*Soonwooa*	0.6 ± 0.3	Clostridiales	2.8 ± 0.2	*Fusobacterium*	1.2 ± 0.1	*Propionibacterium*	1.4 ± 0.3
*Clostridium XlVa*	1.8 ± 0.6	*Prevotella*	0.5 ± 0.3	*Parabacteroides*	2.3 ± 0.2	*Actinomyces*	1.2 ± 0.1	*Corynebacterium*	1.2 ± 0.3
*Enterobacter*	1.7 ± 0.5	*Porphyromonas*	0.5 ± 0.2	*Dialister*	1.7 ± 0.3	*Porphyromonas*	1.0 ± 0.2	Bacillales	0.9 ± 0.0
*Clostridium XVIII*	1.7 ± 0.5	*Actinomyces*	0.4 ± 0.1	*Blautia*	1.3 ± 0.2	Lactobacillales	0.8 ± 0.0	Bacilli	0.6 ± 0.0
*Klebsiella*	1.6 ± 0.5	*Lactobacillus*	0.4 ± 0.2	*Peptoniphilus*	1.2 ± 0.2	*Schlegelella*	0.6 ± 0.1	*Prevotella*	0.6 ± 0.1
*Enterobacteriaceae*	1.5 ± 0.3	*Neisseria*	0.4 ± 0.2	*Campylobacter*	1.2 ± 0.3	—	—	*Actinomyces*	0.5 ± 0.1
*Haemophilus*	1.5 ± 0.5	Bacilli	0.3 ± 0.0	*Anaerosphaera*	1.1 ± 0.2	—	—	*Granulicatella*	0.5 ± 0.1
*Lachnospiraceae*	1.2 ± 0.3	Bacillales	0.3 ± 0.0	*Anaerococcus*	1.1 ± 0.2	—	—	*Clostridium sensu stricto*	0.5 ± 0.1
*Lactococcus*	0.9 ± 0.4	*Pasteurellaceae*	0.2 ± 0.1	*Lactobacillus*	0.9 ± 0.4	—	—	*Lactococcus*	0.3 ± 0.1
*Megasphaera*	0.9 ± 0.4	Actinomycetales	0.2 ± 0.0	—	—	—	—	*Neisseria*	0.2 ± 0.2
*Akkermansia*	0.6 ± 0.6	*Pilibacter*	0.2 ± 0.0	—	—	—	—	*Comamonas*	0.2 ± 0.1
*Prevotella*	0.5 ± 0.3	*Prevotellaceae*	0.2 ± 0.1	—	—	—	—	*Leuconostoc*	0.2 ± 0.1
*Leuconostoc*	0.5 ± 0.2	*Flavobacteriaceae*	0.2 ± 0.0	—	—	—	—	—	—
*Citrobacter*	0.4 ± 0.3	*Bifidobacterium*	0.1 ± 0.1	—	—	—	—	—	—
*Weissella*	0.4 ± 0.2	—	—	—	—	—	—	—	—
*Faecalibacterium*	0.3 ± 0.3	—	—	—	—	—	—	—	—

1 Values are means ± SEM; for infant feces, *n* = 149; infant oral, *n* = 151; maternal feces, *n* = 162; maternal oral, *n* = 182; milk, *n* = 147.

### Relations among complex bacterial memberships over time

**FIGURE 2:**
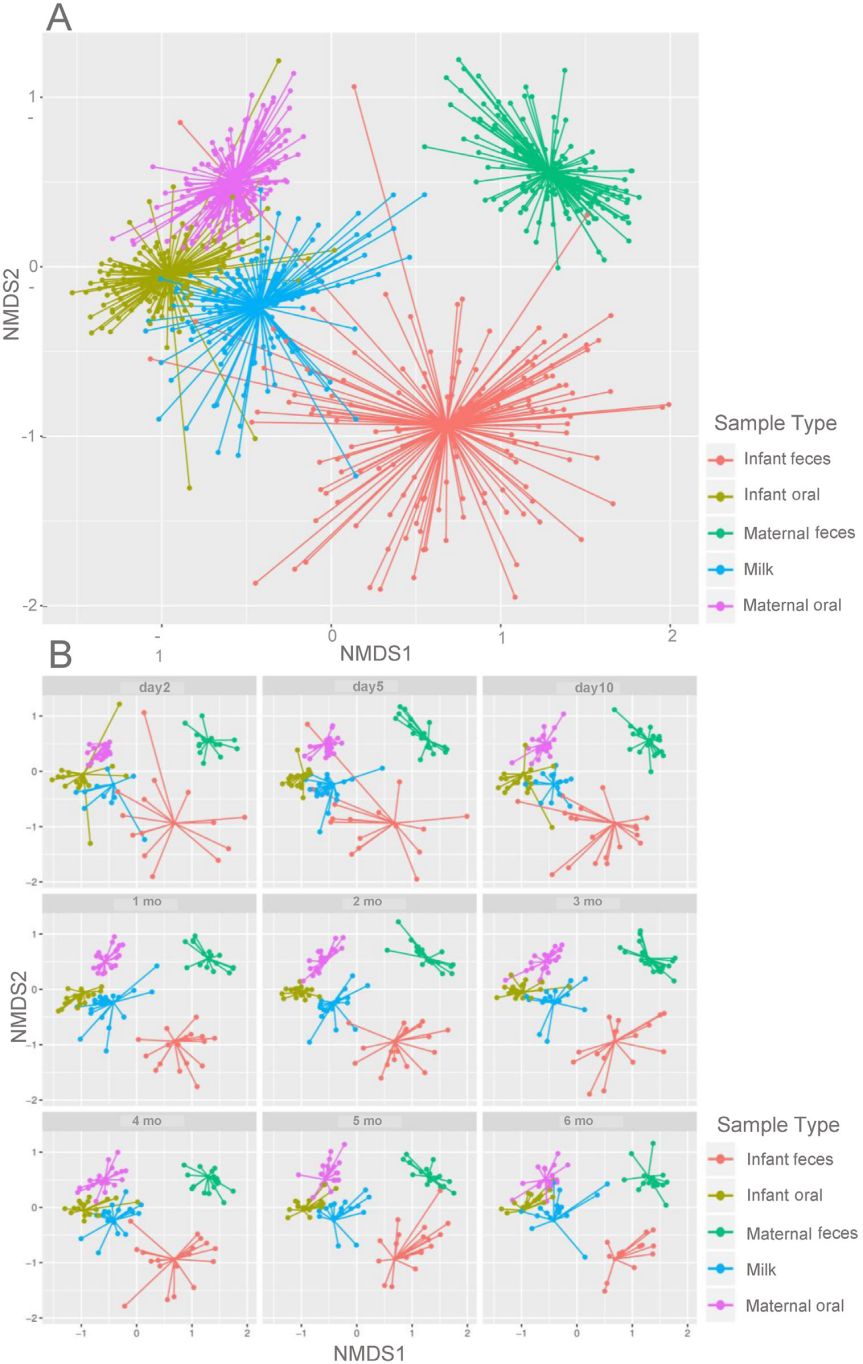
NMDS plots of genus-level (or lowest characterizable level) rarefied sequence read count data from milk, maternal feces, maternal oral swabs, infant feces, and infant oral swabs (A) at all time points combined, and (B) by time postpartum. Each point represents a single sample and is colored by sample type. Segments are drawn connecting each sample to the centroid for the sample type. NMDS, nonmetric multidimensional scaling.

NMDS and PCoA analyses revealed similar results; since the amount of variation explained by the first 2 components of the PCoA at the genus level was only ∼21%, only NMDS results are reported here. There was clustering by sample type despite some overlap (**Figure 2** and **Supplemental Figure 1**). Both ANOSIM (R = 0.61; *P* = 0.001) and adonis (R^2^ = 0.48; *P* = 0.001) tests at the genus level indicated an effect of sample type on complex bacterial community membership, supporting the visual clustering observed in the NMDS plots. NMDS plots suggested that milk and infant fecal samples have some similarity in early life but become increasingly different over time. Conversely, milk and infant oral microbiomes appear to become increasingly similar to each other and to the maternal oral microbiome over time.

### Relations among complex bacterial communities

**FIGURE 3:**
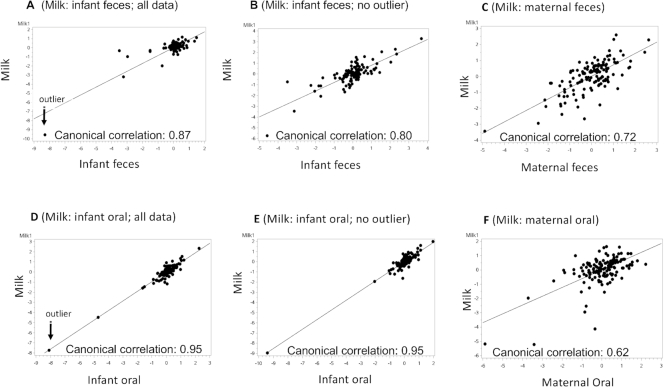
Plots of the first components in each pairwise canonical correlation analysis (with and without outliers when appropriate) between milk and (A, B) infant feces, (C) maternal feces, (D, E) infant oral swabs, and (F) maternal oral swabs. Solid line represents regression line. Each point represents the scores of the linear combinations for a particular sample pair in the canonical correlation.

**FIGURE 4:**
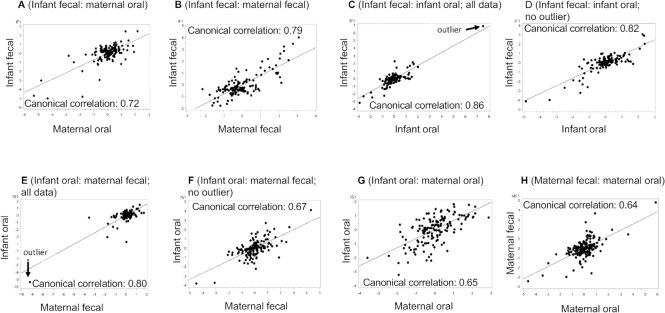
Plots of the first components in each pairwise canonical correlation analysis (with and without outliers when appropriate) between (A) infant feces and maternal oral swabs, (B) infant feces and maternal feces, (C, D) infant feces and infant oral swabs, (E, F) infant oral swabs and maternal feces, (G) infant oral and maternal oral swabs, and (H) maternal feces and maternal oral swabs. Solid line represents regression line. Each point represents the scores of the linear combinations for a particular sample pair in the canonical correlation.

Relations between bacterial communities of the various sample types were further characterized using CCA. Bacterial taxa for each sample type included in the CCA were chosen in accordance with their inclusion in the 10 most abundant taxa for at least 1 time point in that particular sample type. The taxa used for CCA for each sample type are the same as those presented in Table 5. We initially conducted CCA with all data, but in several cases, with single outliers also removed. To visualize the canonical correlations, the first axis scores from each of the sample types included in the correlation were plotted and are illustrated in **Figures 3** and **4**. Each panel displays a canonical correlation plot between different sample types. Each point in a plot represents the scores of the linear combinations for a sample pair in the canonical correlation. The line on the plot represents the linear regression of the points displayed on the plot. For example, Figure 3A displays the results obtained from the CCA of the milk bacteria and the infant fecal bacteria and shows that there is a high correlation (canonical correlation = 0.87) between a linear combination of the relative abundances of the milk bacteria and a linear combination of the relative abundances of the infant fecal bacteria. Figure 3B shows the CCA with the outlier removed.

Most canonical correlations that included all data except 2 (infant oral: maternal oral and milk: maternal oral) had at least 1 significant canonical correlation, all of which were moderate to strong (range: 0.64–0.95; **Table 6**). Two of the canonical correlations (infant feces: infant oral and infant oral: maternal feces) became nonsignificant when the outliers were removed. Milk and infant oral samples had the greatest number of significant canonical components (7 significant components; canonical correlations from 0.95 to 0.70; *P* < 0.003). When all data were included, these 7 canonical components together accounted for ∼91% of the data variability, with the first accounting for ∼31%. Even after removing the outlier, the canonical correlation remained strong (0.95 to 0.66), and we have illustrated relations with and without the outlier (Figure 3D and E, respectively) because we believe that it might be a realistic data point. Milk and infant fecal microbiomes were also highly correlated (Figure 3A), with the first axes accounting for ∼29% of the variation (Table 6). As with the relation between milk and infant oral microbiomes, that between milk and infant feces remained strong (canonical correlation = 0.80; Table 6) with the outlier removed (Figure 3A and B). Milk and maternal feces also had a strong canonical correlation of 0.72 (*P* = 0.0083; Figure 3C); a finding particularly interesting given that their community membership was quite different from each other. The association between infant fecal and maternal fecal bacterial communities was moderately strong (canonical correlation = 0.79, *P* < 0.0001; Figure 4B), as well as the multivariate relation between maternal fecal and maternal oral bacterial communities (canonical correlation = 0.64, *P* < 0.0001; Figure 4H). An overview of the canonical correlations is provided as a heatmap in **Supplemental Figure 2**.

**TABLE 6:** Canonical correlation results for the pairwise comparisons among most abundant bacterial genera in infant feces, infant oral, maternal feces, maternal oral, and milk samples^1^

Relations	Axis	Canonical correlation	Proportion of variability explained	Cumulative variability explained	*P value*
Milk: infant feces (all data)	1	0.87	0.29	0.29	< 0.0001
	2	0.79	0.16	0.46	0.0045
Milk: infant feces (no outlier)	1	0.80	0.22	0.22	0.0107
Milk: infant oral (all data)	1	0.95	0.31	0.31	< 0.0001
	2	0.93	0.23	0.54	< 0.0001
	3	0.90	0.14	0.67	< 0.0001
	4	0.86	0.10	0.77	< 0.0001
	5	0.81	0.07	0.84	< 0.0001
	6	0.73	0.04	0.88	< 0.0001
	7	0.70	0.03	0.91	0.0026
Milk: infant oral (no outlier)	1	0.95	0.33	0.33	< 0.0001
	2	0.93	0.23	0.55	< 0.0001
	3	0.88	0.13	0.68	< 0.0001
	4	0.83	0.08	0.76	< 0.0001
	5	0.76	0.05	0.82	< 0.0001
	6	0.75	0.05	0.87	< 0.0001
	7	0.66	0.03	0.90	0.0122
Milk: maternal feces	1	0.72	0.22	0.22	0.0083
Milk: maternal oral	1	0.62	0.24	0.24	0.33
Infant feces: infant oral (all data)	1	0.86	0.30	0.30	0.0043
Infant feces: infant oral (no outlier)	1	0.82	0.24	0.24	0.10
Infant feces: maternal feces	1	0.79	0.23	0.23	< 0.0001
	2	0.74	0.17	0.40	0.0019
Infant feces: maternal oral	1	0.72	0.27	0.27	0.0005
Infant oral: maternal feces (all data)	1	0.80	0.32	0.32	<0.0001
Infant oral: maternal feces (no outlier)	1	0.67	0.19	0.19	0.15
Infant oral: maternal oral	1	0.65	0.25	0.25	0.18
Maternal feces: maternal oral	1	0.64	0.25	0.25	< 0.0001
	2	0.56	0.16	0.41	< 0.0004
	3	0.55	0.16	0.57	0.0098

1 Milk: infant feces, *n* = 121 pairs; milk: infant oral, *n* = 118 pairs; milk: maternal feces, *n* = 128 pairs; milk: maternal oral, *n* = 142 pairs; infant feces: infant oral, *n* = 121 pairs; infant feces: maternal feces, *n* = 131 pairs; infant feces: maternal oral, *n* = 144 pairs; infant oral: maternal feces, *n* = 136 pairs; infant oral: maternal oral, *n* = 147 pairs; maternal feces: maternal oral, *n* = 159 pairs.

### Potential sources of the bacterial communities in milk and infant feces

Using SourceTracker2 software, we first estimated likely contributions to the infant fecal bacterial communities (sink) using rarefied taxon read counts at the genus level from milk (source), infant oral (source), maternal oral (source), and maternal fecal (as a proxy of the maternal GI bacteria; source). The milk microbiome was estimated to directly contribute ∼4.9% at day 2 and ∼0.3% at month 6 to the infant fecal microbiome (**Table 7**). Note however, a large percentage (87–98%) was unknown, and it is plausible that the milk contributes to the microbiomes present in the stomach, small intestine, and upper large intestine, which consequently help shape that of the colon and feces. To estimate the potential sources of the milk bacterial communities (sink), infant oral (source), maternal oral (source), and maternal fecal (source) rarefied taxon read counts at the genus level were used. Infant oral bacterial communities were estimated to contribute ∼21% at day 2 and then up to 66% at month 5 (**Table 8**). Maternal oral bacterial communities contributed 26% at day 2 and then only 2–6% from 1–6 mo postpartum.

**TABLE 7:** Relative amounts (% of total) that bacterial communities found in infant oral, maternal feces, maternal oral, and milk may contribute to the bacterial composition of infant feces from day 2 to 6 mo of life^1^

	d 2	d 5	d 10	1 mo	2 mo	3 mo	4 mo	5 mo	6 mo
Infant oral	1.1 ± 0.3	1.9 ± 0.5	2.0 ± 0.9	0.7 ± 0.2	0.9 ± 0.6	0.5 ± 0.2	3.1 ± 2.5	0.7 ± 0.3	0.2 ± 0.1
Maternal feces	0.9 ± 0.3	0.6 ± 0.2	0.5 ± 0.2	0.7 ± 0.2	0.6 ± 0.1	0.6 ± 0.1	0.8 ± 0.2	2.8 ± 2.0	0.7 ± 0.2
Maternal oral	6.2 ± 3.5	8.1 ± 5.6	9.1 ± 5.1	0.4 ± 0.1	0.3 ± 0.1	0.3 ± 0.1	0.5 ± 0.2	0.3 ± 0.1	0.2 ± 0.1
Milk	4.9 ± 3.3	1.2 ± 0.3	1.1 ± 0.4	0.4 ± 0.1	0.4 ± 0.1	0.4 ± 0.1	0.5 ± 0.2	0.5 ± 0.1	0.3 ± 0.1
Unknown	87.0 ± 6.8	88.2 ± 6.0	87.3 ± 6.0	97.8 ± 0.4	97.9 ± 0.8	98.2 ± 0.3	95.1 ± 2.8	95.7 ± 2.0	98.5 ± 0.2

1 Values are means ± SEM; infant feces, *n* = 149; infant oral, *n* = 151; maternal feces, *n* = 162; maternal oral, *n* = 182; milk, *n* = 147.

**TABLE 8:** Relative amounts (% of total) that bacterial communities found in infant oral, maternal feces, and maternal oral may contribute to the bacterial composition of milk from day 2 to 6 mo of life^1^

	d 2	d 5	d 10	mo 1	mo 2	mo 3	mo 4	mo 5	mo 6
Infant oral	21.1 ± 9.2	42.6 ± 7.8	39.8 ± 8.5	56.7 ± 8.5	65.3 ± 7.0	65.2 ± 7.9	55.8 ± 8.8	66.3 ± 7.2	50.0 ± 8.3
Maternal feces	0.3 ± 0.1	0.6 ± 0.3	0.6 ± 0.2	0.6 ± 0.1	0.7 ± 0.2	0.9 ± 0.4	1.2 ± 0.5	1.8 ± 0.7	1.0 ± 0.4
Maternal oral	26.0 ± 7.7	10.8 ± 3.3	15.9 ± 4.8	2.1 ± 0.3	2.8 ± 0.4	2.6 ± 0.3	2.8 ± 0.4	3.8 ± 0.6	6.7 ± 2.4
Unknown	52.6 ± 9.2	46.1 ± 8.8	43.6 ± 9.4	40.7 ± 8.6	31.2 ± 7.1	31.3 ± 8.0	40.2 ± 9.1	28.0 ± 7.2	42.3 ± 8.3

1 Values are means ± SEM; infant feces, *n* = 149; infant oral, *n* = 151; maternal feces, *n* = 162; maternal oral, *n* = 182; milk, *n* = 147. d, day; mo, month.

## Discussion

This study relates and compares the membership and composition of microbiomes from maternal milk, feces, and oral samples, and infant feces and oral samples from the same mother-infant dyad at 9 time points in the first 6 mo postpartum. Samples from the different niches showed distinct clustering at the genus level as has been shown previously for other subsets of mother-infant pairs (21–24). Bacterial communities from each site also reflected those previously reported for maternal oral (21, 24), fecal (2, 24, 26, 44), and milk (20–23, 45). Our results for the infant fecal microbiome generally concurred with previous reports (17, 46). However, we did not observe an increase in alpha diversity over time in the infant fecal microbiome as reported in some previous studies (17, 22) but this may be due to the smaller sample size in our cohort. We also observed a lower relative abundance of Bifidobacterium than other studies (∼5.4% compared with ∼20.2% and ∼30%, respectively; 17, 22). Our results also concur with previous studies on the infant oral microbiome, which was generally predominated by *Streptococcus* (21, 23, 24, 47, 48) and relatively high proportions of *Staphylococcus*. This is similar to work performed by Costello and coworkers (16) in which they also observed high relative abundances of *Staphylococcus* in oral samples collected from a small group of low-birthweight infants. We posit that the high relative abundance of *Staphylococcus* in the infant's mouth is likely associated with the high proportions of *Staphylococcus* in milk and on the mother's skin. With the frequent bi-directional interaction between the mammary gland and the infant's mouth during breastfeeding (49), it is not surprising to find similar bacteria in milk and infant oral swabs.

Our data do not support our a priori hypothesis that the milk microbiome would most closely resemble that of infant feces. In fact, we observed that the milk microbiome was more similar to that of infant oral and, as time progressed, became more similar to both infant and maternal oral microbiomes. These results concur with previous studies (21–23) and provide evidence that this trend continues until at least 6 mo. Nonetheless, even though the microbiomes (e.g., types of bacteria present) were different between milk and infant feces, CCA suggests that these two bacterial communities are intimately linked. Similarly, although the bacterial composition of maternal feces was distinct from that in milk, there was a strong canonical correlation between them. This provides evidence that the maternal GI microbial communities and/or factors such as maternal diet may influence the milk microbiota.

There are several important limitations to this study. Although our sample size was larger than that of some others (20, 21), it was smaller than some (22–25, 28) and represented a small geographic area. Previous evidence has indicated that the microbiomes of the GI tract, oral, and milk may differ based on geography, ethnicity, social networks, and/or diet (50–55). Future studies should investigate if and how the microbiomes of maternal-infant dyads in different populations are related to each other. In addition, insufficient statistical power does not allow us to address the influence of other factors such as mode of delivery, maternal intrapartum antibiotic prophylaxis, probiotic usage, and supplemental feedings to the infant. However, these factors need to be examined in light of previous work that suggest they may influence maternal GI (56), infant GI (57–59), infant oral (60), and milk (61–63) microbial compositions. Another limitation is that only one sample for each of the sample types was collected from the mother-infant dyad at each time point. It is unclear as to the extent of day-to-day or even within-day variation that might exist in each of the different sample types, and little data exist describing this variation. Koenig and coworkers (64) conducted a case study focusing on infant feces, in which they performed extensive sampling of the fecal microbiome in one infant from birth through 2.5 y of life. They observed considerable variation in the composition of the fecal microbiome across the span of 2–4 d. However, they also observed a temporal gradient in the community characteristics that would have been captured in our sampling scheme. For milk, we have previously shown that the milk microbiome appears to remain relatively stable across 5 wk during mid-lactation (45), but to our knowledge no study has reported daily or even within-a-day variation of the human milk microbiome. Lazarevic and coworkers (65) investigated the inter- and intra-individual variation in the salivary microbiomes from 5 adults. They concluded that the oral microbiome appeared to be stable over at least 5 d and seemed to remain stable across longer spans of time. This is similar to our findings that there is little to no change in the most abundant taxa in the maternal oral microbiome. There are also methodological limitations that should be considered such as possible biases derived from the slightly different protocols used for DNA extraction and PCR amplification. Bacterial concentrations in milk from “healthy” lactating women are also low (66) and thus, bacterial DNA profiles from milk samples might be more susceptible to “contaminating DNA” (67, 68). It should be noted that we implemented negative controls during DNA extractions and PCR, and found no evidence of contamination; however, we did not sequence the negative controls. Future studies should consider sequencing the negative controls and the appropriate use of these in downstream analyses. Another methodological limitation is that 5 MiSeq runs were conducted over the course of 3 y. Each sample type was sequenced on a different sequencing run on the same machine, but we did not implement any inter-assay sequencing controls; thus, we are unable to adjust for potential batch effects. It is noteworthy that, once all samples were sequenced, data from all runs were processed together using the same software and *16S* rRNA database. Different sample types also had different average yields of sequence reads. In order to minimize the bias of having large variation in the sequencing depth between samples and sample types, we rarefied the read counts to a minimum threshold of 2,000 reads. Although we recognize that rarefying results in a loss of information, this approach has been suggested (69) to help lower the false discovery rate when large differences in average library size exist among groups. Another limitation in the study is that bacterial communities were characterized using only the targeted sequencing of the V1-V3 hypervariable region of the *16S* rRNA gene. Therefore, we could not discern whether the DNA was from live or dead bacteria and were only able to characterize the majority of the sequence reads to the genus level. The results presented are also compositional in nature. Recently, Gloor and coworkers (70) reviewed the inherent limitations of compositional data and thus, caution must be used in interpreting the data presented here. Future longitudinal studies that both enumerate and classify bacteria to the species- or strain-level as well as determine the viability of the bacteria present will provide greater understanding as to the intricate interactions that may occur between bacterial communities of the mother and her infant. Finally, although our findings suggest that maternal and infant oral microbiomes are interconnected with the milk microbiome, intervention studies will be needed to confirm the directionality of these relations, should they turn out to be causal in nature.

In conclusion, microbial communities of the mother-infant epi-holobiont represent complex, collective microcosms that likely interact to maintain health in both mothers and infants (12, 71–73). The close proximity and interchange that occurs between mothers and their infants, particularly during breastfeeding, possibly influence bacterial communities of both. Understanding these complex cross-biome interactions is important as establishment of these different microbiota in various body habitats potentially has consequences for acute and chronic health of both the mother and infant.

## Supplementary Material

nxy299_Supplemental_Files

## Notes

Supported by grants from the Bill and Melinda Gates Foundation; the National Institute of General Medical Sciences from the National Institutes of Health, P30 GM103324, and the USDA National Institute of Food and Agriculture (Hatch) through the Idaho Agricultural Experiment Station.

Author disclosures: JEW, JMC, KAL, NFB, SLB, HKP, KMS, MAY, BS, WJP, MAM, and MKM, no conflicts of interest.

Supplemental Table 1 and Supplemental Figures 1 and 2 are available from the “Supplementary data” link in the online posting of the article and from the same link in the online table of contents at https://academic.oup.com/jn/.

Abbreviations used: CCA, canonical correlation analysis; GI, gastrointestinal; GLMM, generalized linear mixed model; NMDS, nonmetric multidimensional scaling; PCoA, principal coordinates analysis; RDP, Ribosomal Database Project; rRNA, ribosomal RNA; TE, Tris-EDTA.
