# Supplementary material for: Strong Multivariate Relations Exist Among Milk, Oral, and Fecal Microbiomes in Mother-Infant Dyads During the First Six Months Postpartum
Source: J Nutr. 2019 May 7;149(6):902–14. doi: 10.1093/jn/nxy299 (PMC6543206; doi:10.1093/jn/nxy299)
Supplement: nxy299_Supplemental_Files [file nxy299_supplemental_files.zip › Supplemental_Table_1_11-2-2018.pdf]

Supplementary data

Supplemental Table 1. *Overall relative abundances of the 20 most abundant genera (or next highest characterizable taxa) in infant feces, infant oral, maternal feces, maternal oral, and milk samples averaged over all timepoints.*[1]

Infant feces	%	Infant oral	%	Maternal feces	%	Maternal oral	%	Milk	%
Bacteroides	21.4 ± 2.4	*Streptococcus*	69.1 ± 1.8	*Bacteroides*	22.9 ± 1.3	*Streptococcus*	53.9 ± 1.3	*Streptococcus*	47.1 ± 2.3
Escherichia Shigella	16.0 ± 2.0	*Gemella*	9.5 ± 1.3	*Faecalibacterium*	8.8 ± 0.6	*Rothia*	6.3 ± 0.5	*Staphylococcus*	24.1 ± 2.2
Veillonella	10.5 ± 1.4	*Rothia*	5.7 ± 1.0	*Prevotella*	7.6 ± 1.0	*Gemella*	6.2 ± 0.5	*Gemella*	3.6 ± 0.8
Clostridium sensu stricto	9.0 ± 1.6	*Staphylococcus*	4.1 ± 0.9	*Lachnospiracea incertae sedis*	7.6 ± 0.4	*Prevotella*	5.8 ± 0.5	*Rothia*	2.6 ± 0.5
Bifidobacterium	5.4 ± 0.9	*Veillonella*	2.6 ± 0.3	*Lachnospiraceae*	5.5 ± 0.3	*Veillonella*	5.3 ± 0.3	*Veillonella*	2.5 ± 0.3
Streptococcus	4.6 ± 1.2	Lactobacillales	1.2 ± 0.0	*Porphyromonas*	4.2 ± 0.6	*Neisseria*	4.5 ± 0.4	*Lactobacillus*	1.7 ± 0.7
Parabacteroides	4.0 ± 1.0	*Granulicatella*	1.1 ± 0.2	*Ruminococcaceae*	3.4 ± 0.2	*Granulicatella*	2.7 ± 0.2	*Pseudomonas*	1.7 ± 0.6
Lachnospiracea incertae sedis	2.8 ± 0.7	*Haemophilus*	0.9 ± 0.4	*Oscillibacter*	3.2 ± 0.3	*Haemophilus*	2.7 ± 0.2	Lactobacillales	1.7 ± 0.1
Pseudomonas	2.7 ± 0.5	*Soonwooa*	0.6 ± 0.3	Clostridiales	2.8 ± 0.2	*Fusobacterium*	1.2 ± 0.1	*Propionibacterium*	1.4 ± 0.3
Clostridium XIVa	1.8 ± 0.6	*Prevotella*	0.5 ± 0.3	*Parabacteroides*	2.3 ± 0.2	*Actinomyces*	1.2 ± 0.1	*Corynebacterium*	1.2 ± 0.3
Enterobacter	1.7 ± 0.5	*Porphyromonas*	0.5 ± 0.2	*Dialister*	1.7 ± 0.3	*Porphyromonas*	1.0 ± 0.2	Bacillales	0.9 ± 0.0
Clostridium XVIII	1.7 ± 0.5	*Actinomyces*	0.4 ± 0.1	*Roseburia*	1.5 ± 0.1	Lactobacillales	0.8 ± 0.0	Bacilli	0.6 ± 0.0
Klebsiella	1.6 ± 0.5	*Lactobacillus*	0.4 ± 0.2	*Blautia*	1.3 ± 0.2	*Prevotellaceae*	0.8 ± 0.1	*Prevotella*	0.6 ± 0.1
Enterobacteriaceae	1.5 ± 0.3	*Neisseria*	0.4 ± 0.2	*Clostridium.XIVa*	1.2 ± 0.2	*Leptotrichia*	0.7 ± 0.1	*Actinomyces*	0.5 ± 0.1
Haemophilus	1.5 ± 0.5	Bacilli	0.3 ± 0.0	*Peptoniphilus*	1.2 ± 0.2	*Schlegelella*	0.6 ± 0.1	*Granulicatella*	0.5 ± 0.1
Lachnospiraceae	1.2 ± 0.3	Bacillales	0.3 ± 0.0	*Campylobacter*	1.2 ± 0.3	*Neisseriaceae*	0.5 ± 0.0	Actinomycetales	0.5 ± 0.1
Lactococcus	0.9 ± 0.4	*Pasteurellaceae*	0.2 ± 0.1	*Anaerosphaera*	1.1 ± 0.2	*Abiotrophia*	0.3 ± 0.1	*Clostridium sensu stricto*	0.5 ± 0.1

Supplementary data

Megasphaera	0.9 ± 0.4	Actinomycetales	0.2 ± 0.0	*Anaerococcus*	1.1 ± 0.2	*Pasteurellaceae*	0.3 ± 0.0	Firmicutes	0.5 ± 0.0
Akkermansia	0.6 ± 0.6	*Pilibacter*	0.2 ± 0.0	*Alistipes*	1.1 ± 0.1	Actinomycetales	0.2 ± 0.0	*Staphylococcaceae*	0.5 ± 0.0
Sutterella	0.6 ± 0.2	Firmicutes	0.2 ± 0.0	*Finegoldia*	1.0 ± 0.2	*Soonwooa*	0.2 ± 0.0	*Bacteroides*	0.4 ± 0.1

[1]Values are means ± SEM; infant feces, *n* =149; infant oral, *n* = 151; maternal feces, *n* = 162; maternal oral, *n* = 182; milk, *n* = 147.
