# Supplementary material for: Strong Multivariate Relations Exist Among Milk, Oral, and Fecal Microbiomes in Mother-Infant Dyads During the First Six Months Postpartum
Source: J Nutr. 2019 May 7;149(6):902–14. doi: 10.1093/jn/nxy299 (PMC6543206; doi:10.1093/jn/nxy299)
Supplement: nxy299_Supplemental_Files [file nxy299_supplemental_files.zip › Supplemental_Figure_1.pdf]

Supplementary data

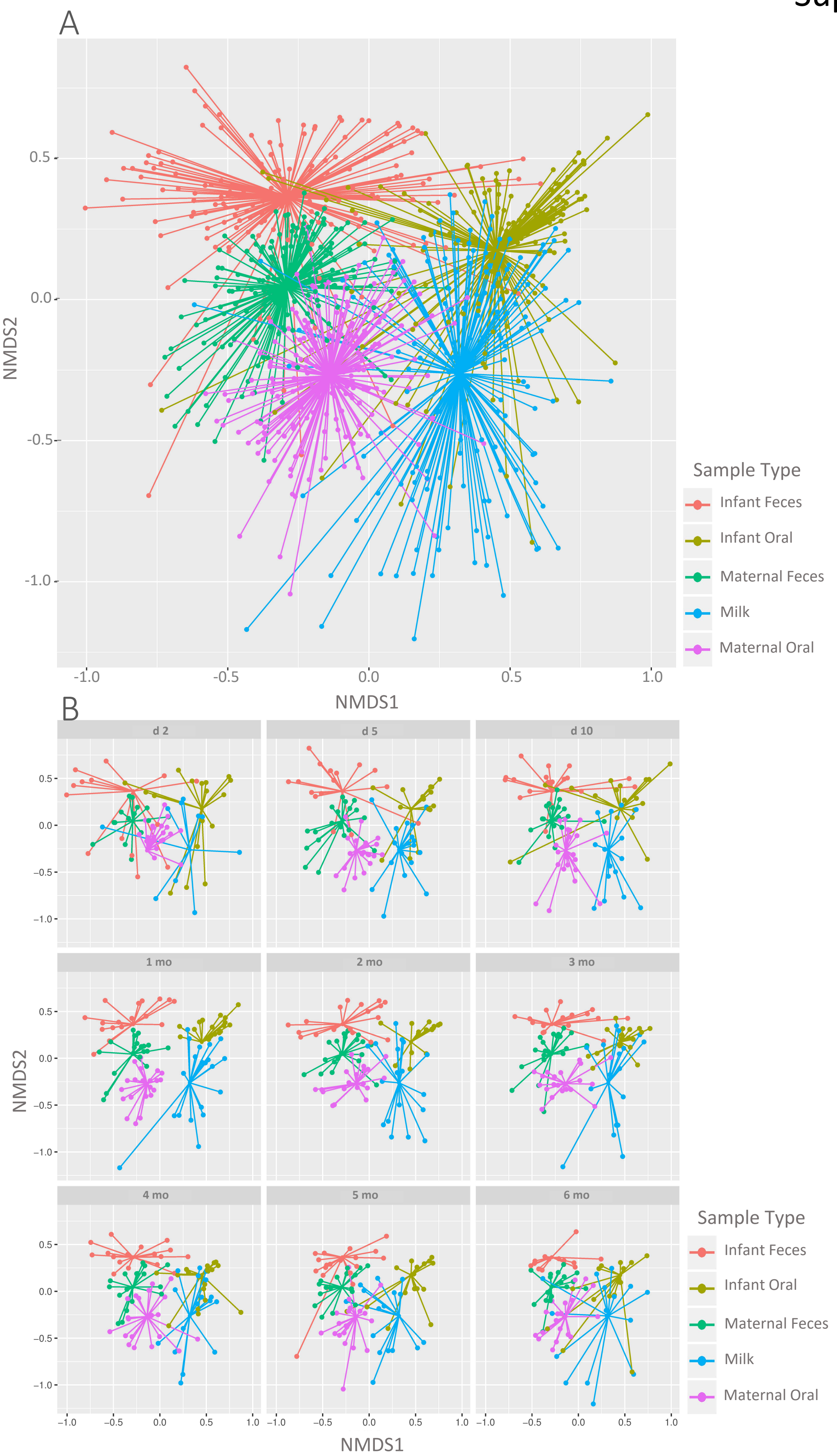

Supplementary Figure 1 – NMDS plots of phylum-level rarefied sequence read count data from milk , maternal feces, maternal oral swabs, infant feces, and infant oral swabs (A) at all timepoints combined, and (B) by time postpartum. Each point represents a single sample and is colored by sample type. Segments are drawn connecting each sample to the centroid for the sample type.
