# Supplementary material for: Strong Multivariate Relations Exist Among Milk, Oral, and Fecal Microbiomes in Mother-Infant Dyads During the First Six Months Postpartum
Source: J Nutr. 2019 May 7;149(6):902–14. doi: 10.1093/jn/nxy299 (PMC6543206; doi:10.1093/jn/nxy299)
Supplement: nxy299_Supplemental_Files [file nxy299_supplemental_files.zip › Supplemental_Figure_2.pdf]

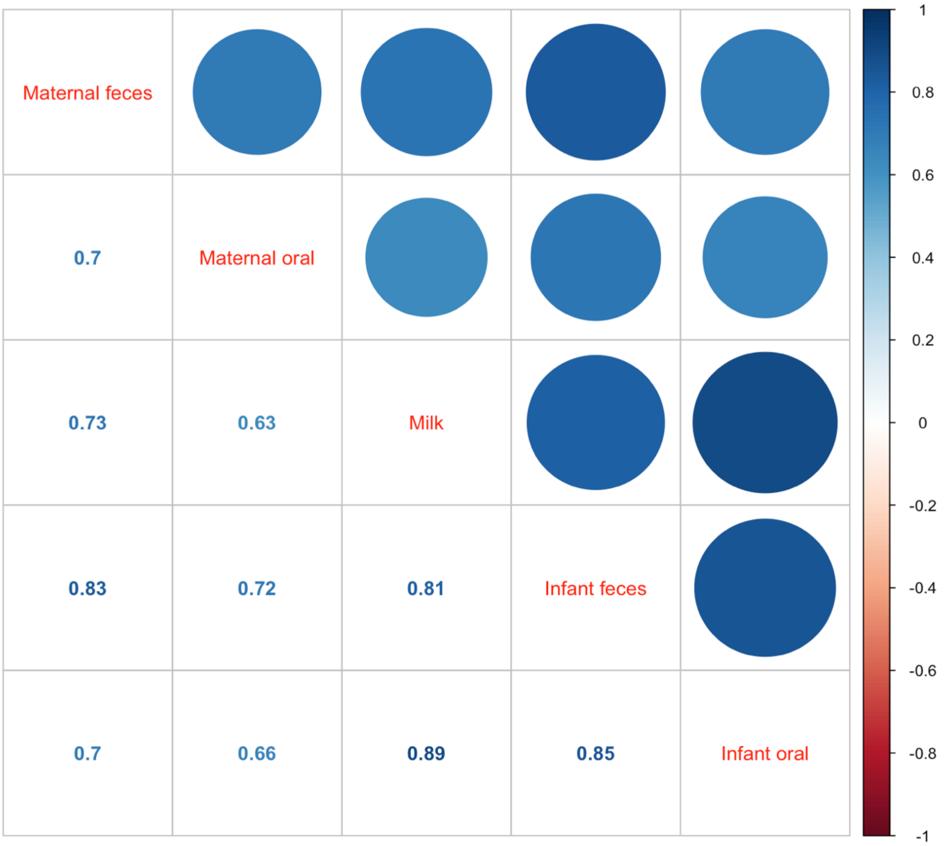

Supplemental Figure 2 – Canonical correlations between sample types. Size and color of circles indicate strength of correlation; values are the canonical correlations.
